# Effects of a 12-Week Modified German Volume Training Program on Muscle Strength and Hypertrophy—A Pilot Study

**DOI:** 10.3390/sports6010007

**Published:** 2018-01-29

**Authors:** Daniel A. Hackett, Theban Amirthalingam, Lachlan Mitchell, Yorgi Mavros, Guy C. Wilson, Mark Halaki

**Affiliations:** Discipline of Exercise and Sport Science, The University of Sydney, Sydney, NSW 2141, Australia; thebanamir93@hotmail.com (T.A.); lachlan.mitchell@sydney.edu.au (L.M.); yorgi.mavros@sydney.edu.au (Y.M.); guy.wilson@sydney.edu.au (G.C.W.); mark.halaki@sydney.edu.au (M.H.)

**Keywords:** resistance training, muscle hypertrophy, muscle strength, training volume

## Abstract

This study investigated the effect of a 12-week modified German Volume Training intervention, or the 10 sets method, on muscle strength and hypertrophy. Twelve healthy males were randomly assigned to either a 5-SET or 10-SET group and performed 5 or 10 sets, respectively, of 10 repetitions at 60–80% one-repetition maximum (1RM). Muscle strength and body composition measures were taken at baseline, six weeks, and after 12 weeks of training. No significant changes in total, trunk, and arm lean mass were found within and between groups at any time point. There was no significant difference between groups for lean leg mass. However, a decrease in lean leg mass was observed within the 10-SET group between six and 12 weeks (*p* = 0.02). An increase in 1RM bench press was found within the 5-SET group at week 6 (*p* = 0.001) and 12 (*p* = 0.001) when compared to baseline, while no increases in 1RM leg press were observed at any time point within any group. No significant differences were found for 1RM bench press and leg press between groups. For 1RM bench press moderate effect sizes (ES) favored 5-SET and for 1RM leg press small ESs favored 10-SET. Findings suggest performing >5 sets per exercise does not promote greater gains in muscle strength and hypertrophy. Future research should aim to substantiate these preliminary findings in a larger cohort.

## 1. Introduction

Resistance training is a physical activity that is commonly used to develop muscle strength and stimulate muscle hypertrophy. Maximizing these training adaptations involves the appropriate manipulation of resistance training variables [1]. Arguably, one of the most critical variables influencing the effectiveness of resistance training on muscle strength and hypertrophy is volume [2]. Resistance training volume is defined as the total number of repetitions (repetitions x sets) together with the loads used for a given exercise. However, previous studies examining the effect of resistance training volume on muscle adaptations have done so via controlling for factors that influence intensity (i.e., repetitions and load) and only manipulating the sets performed [3,4,5,6,7]. Based on current resistance training guidelines for muscle strength and hypertrophy, it is recommended that untrained individuals perform a lower number of sets per exercise compared to advanced trainers (1–3 sets compared 3–6 sets, respectively) [8]. These guidelines suggest that as training experience increases, muscle strength and hypertrophy gains are optimized with an increased volume of up to six sets per exercise.

Three systematic reviews and meta-analyses have provided some interesting insights into the effects of set number on muscle strength and hypertrophy [2,9,10]. Krieger [9,10] found that 40% greater muscle strength and hypertrophy gains can be achieved with 2–3 sets compared to a single set per exercise. Furthermore, Krieger [9,10] showed a dose-response relationship with greater muscle strength and hypertrophy gains with an increased number of sets, up to approximately 4–6 sets, where no further gains were observed. These findings support the recommended set range for advance trainers from the resistance training guidelines [8]. Also, Schoenfeld et al. [2] analyzed the impact of the total number of weekly sets per muscle group which was suggested to be a more relevant marker of training volume. The results of this review and meta-analysis indicated a dose-response relationship between weekly resistance training volume and muscle mass, and it was concluded that at least 10 weekly sets per muscle group is necessary to maximize muscle mass.

It is well known that mechanical loading stimulates protein synthesis in skeletal muscle and lifting heavier loads will increase this response until a plateau occurs [11]. However, despite evidence for a plateau of muscle strength and hypertrophy gains beyond 4–6 sets [9,10], the upper threshold for number of sets still remains unclear. It has been suggested that a large resistance training volume (i.e., greater set number) will induce extensive metabolic stress and mechanical tension that leads to greater substrate depletion, metabolite accumulation and muscle damage [12]. With adequate post-training recovery, these factors will promote anabolic responses that lead to enhancing muscle mass [13] and possibly strength [14,15]. However, resistance training with volumes too far beyond an “upper threshold” will likely be counterproductive for strength-related tasks in general and may mute or debilitate the hypertrophic response [16].

German Volume Training (GVT) is a practice that has been used by national weightlifting coaches to increase muscle mass of their athletes in the off-season [17]. A typical GVT session involves performing 10 sets of 10 repetitions (i.e., 100 repetitions) for two compound resistance exercises at loads of ~60% 1RM or 20RM [18]. Together with this high training volume, the recovery between sets is relatively short (~60–90 s) to induce greater metabolic stress (e.g., buildup of metabolites such as lactate). We recently examined the effectiveness of a modified version of the GVT program (10 sets method) compared to the upper end of the set range commonly used by resistance trainers (five sets) over a duration of six weeks. Briefly, the modification to the traditional GVT program included the performing of assistive exercises following completion of two exercises performed for 10 sets. Also, squats and deadlifts are traditionally used in GVT but were changed to leg press and lunges. The results from the previous study showed that no additional gains in muscle hypertrophy can be achieved when following a modified version of the GVT program compared to training with five sets over a duration of six weeks [19]. 

Another interesting finding from our previous study was greater increases in upper body strength for five sets compared to 10 sets, which is different to the few studies that have investigated the effects of a similar number of sets on muscle strength. Drinkwater et al. [20] found no differences in bench press one-repetition maximum (1RM) following a six-week bench press training program comparing eight sets versus 12 sets. Likewise, Marshall et al. [6] found no differences in squat 1RM when comparing four sets to eight sets following a six-week squat training program, although a significant increase in squat 1RM was observed for eight sets compared to one set. However, due to the relatively short duration of our previous study (i.e., six weeks) [19] as well as the other studies noted above, it is unknown whether this may have affected the results. For instance, Stark et al. [21] concluded that a resistance training protocol tailored for muscle strength and hypertrophy should be at least 10–12 weeks duration involving 3–5 sessions per week. Therefore, the effectiveness of a modified GVT program (i.e., 10 sets method) on muscle strength and hypertrophy when performed over a longer period of time is unknown. 

The aim of this study was to investigate the effects of a 12-week training program involving five sets (5-SET) versus 10 sets (10-SET) of resistance exercises on muscle strength and hypertrophy. Only the sets performed for the first two exercises in each training session were manipulated, while the sets performed for all other exercises for the two groups were the same. Additionally, both groups were prescribed the same initial loads for each exercise in an effort to isolate the effect of an increased number of sets on the outcome measures. It was hypothesized that greater muscle hypertrophy would be observed for 10-SET compared to 5-SET following 12 weeks training. It was also hypothesized there would be no differences in muscle strength between groups following the resistance training intervention.

## 2. Methods

### 2.1. Participants

Twelve healthy males were randomly assigned to either a 5-SET group (*n* = 6; age = 23.7 ± 3.0 years; body mass = 76.0 ± 16.4 kg; height = 180.7 ± 6.0 cm) or 10-SET group (*n* = 6; age = 23.6 ± 2.9 years; body mass = 83.3 ± 7.1 kg; height = 176.4 ± 13.0 cm). Participants had a minimum of one year of resistance training experience at the recreational level and had been performing at least three resistance training sessions per week consistently over the previous three months. All participants reported not having used anabolic steroids or any other legal or illegal agents known to increase muscle size during the previous year. Written informed consent was obtained from all participants before commencing the study. Participants were asked to refrain from any resistance training away from the supervised sessions. The study was approved by the University of Sydney Human Research Ethics Committee.

### 2.2. Experimental Design

This study was carried out over a period of 12 weeks to determine the effects of performing five sets versus 10 sets of compound resistance exercises on muscular hypertrophy and strength. Outcomes were measured at baseline, six weeks, and 12 weeks. Training was performed three times per week, with all sessions supervised by fourth-year university-trained students from the Discipline of Exercise and Sport Science. Whey protein was consumed within 30 min post-exercise to aid in potentiation of muscle protein synthesis following training sessions.

### 2.3. Resistance Training

The training program consisted of a “split-routine” that involved performing different exercises targeting specific muscle groups during the three training sessions per week. The chest and upper back were trained in session 1, the legs in session 2, while the shoulders and arms were trained in session 3. During each training session, the 5-SET and 10-SET groups performed the same exercises (Table 1). The only difference between the intervention groups was the number of sets performed for the first two compound exercises during each training session (e.g., five versus 10 sets for the bench press). Exercises (with the exception of abdominal exercises and calf raisers) were performed for 10 repetitions at loads of 60–80% 1RM with 60–90 s recovery between sets. If 10 repetitions could not be performed during an exercise with a given load the participants were instructed to perform as many repetitions possible. Therefore, the loads lifted for exercises during sessions were not reduced if participants could not complete 10 repetitions. On the last set for all exercises participants were instructed to perform repetitions to the point of momentary failure (i.e., no longer able to produce adequate force to lift the load and complete the repetition). During all sets, repetitions were performed in a controlled manner (~1 s concentric contraction and ~2 s eccentric contraction). When participants were able to complete >10 repetitions on the final set and 10 repetitions for the previous sets of an exercise (with correct technique), the training load was increased by approximately 5–10%. The increase in training loads were influenced by the exercise, with generally greater increases in load for exercises involving larger compared to smaller muscle groups.

### 2.4. Body Composition

A whole-body dual energy x-ray absorptiometry scanner (Lunar Prodigy, GE Medical Systems, Madison, WI, USA) was used to measure body composition. Scans were performed under standardized conditions (early morning, overnight fasted, and standardized body positioning on the scanning bed) by two licensed co-investigators (Yorgi Mavros and Guy C. Wilson). Inter-rater reliability based on scans at baseline was excellent for lean body and fat mass (intraclass correlation coefficient: 0.98–0.99 and coefficient of variation: 1.1–2.4%, respectively). Total and regional lean tissue and fat mass were determined using the system’s software package enCORE 2011 (version 13.60.033, GE Healthcare Lunar, Madison, WI, USA). 

### 2.5. Maximal Strength

The one-repetition maximum (1RM) test was used to assess maximal strength for the flat barbell bench press and horizontal leg press. Participants refrained from any exercise other than activities of daily living for at least 48 h prior to 1RM testing. Prior to assessing the 1RM, a specific warm-up was performed involving a set of five repetitions at ~50% of perceived 1RM followed by 1–2 sets of 2–3 repetitions at a load corresponding to ~60–80% 1RM. The 1RM protocol involved performing trials of a single repetition of increasing load (~5–10% increments) with 3–5 min rest between attempts. This cycle was continued until the participant was unable to complete a lift, with the 1RM being the heaviest load that was successfully lifted. A successful bench press attempt was achieved if the barbell was lowered close to the chest, followed by lifting the bar until the elbows were almost straight. Additionally, for a successful 1RM bench press, participants needed to display minimal back arching and swaying. For the leg press, participants commenced with their knees flexed to approximately 90°. A successful 1RM leg press attempt was achieved through extension of the hips and knees until almost straight and then returning to the starting position. The test-retest intraclass correlation coefficient from our laboratory for 1RM bench press and leg press were ≥0.90. 

### 2.6. Diet

Throughout the study, participants were encouraged to increase their caloric intake. To acquire the extra amount of calories, participants were advised to eat slightly larger portions during each meal of their usual diet and avoid taking any supplements other than that provided in the course of the study. To maximize the potential for muscle strength and lean mass gains as a result of training, participants consumed a protein supplement within 30 min post-exercise, as is usually practiced by experienced resistance trainers. The supplied supplement (Venom whey protein concentrate; The Ausray Group, Queensland, Australia) contained 30.8 g protein, 0.2 g fat, and 0.9 g carbohydrate. 

### 2.7. Statistical Analyses 

All analyses were performed using SPSS version 22.0 for Windows (IBM Corp., Armonk, NY, USA). An independent t-test was used to compare baseline characteristics (body composition and muscular strength) and the training variables (% 1RM and volume load) of 5-SET and 10-SET groups over the 12 weeks. A 2 × 3 (group × time) analysis of variance was used to examine differences between training protocols (5-SET versus 10-SET) on body mass, body mass index (BMI), body fat (%), lean mass (total body, trunk, arm, and leg), and strength (1RM bench press and leg press). Tukey post hoc tests were used when significant analysis of variance results were found. Data are presented as means ± standard deviation (SD). The level of significance was set at *p* < 0.05 and trends were declared at *p* = 0.05 to <0.10. Furthermore, 95% confidence intervals (CI) were calculated in addition to effect size (ES) using Cohen’s *d* [22] for each outcome to determine the magnitude of differences found within and between each training condition. The ES were calculated from the difference between mean post-test scores and dividing by pooled SD.

An ES of 0.20 or less was considered a trivial effect, 0.21 to 0.59 a small effect, 0.60 to 1.19 a moderate effect, 1.20 to 1.99 a large effect, 2.0 to 3.9 a very large effect, and >4.0 a nearly perfect effect [23]. Due to the relatively small number of participants recruited for the present study (*n* = 12), when there were ESs > 0.20 between groups that were not statistically significant (*p* > 0.05), the total sample size required to reach statistical significance (power of 95%) was determined using G*power 3.1.5 [24].

## 3. Results

Overall attendance was good, with a mean participation rate of 93%, and there were no adverse events as a result of the exercise intervention. There were no significant differences between groups at baseline for muscle strength (1RM bench press and leg press) and body composition (body mass, BMI, body fat (%), total body lean mass, lean trunk mass, lean arm mass, and leg lean mass). The average total volume load (load × repetitions) per session was significantly greater for the 10-SET compared to the 5-SET group (*p* = 0.001). The relative training intensity (% 1RM) increased over the 12 weeks for the bench press (*p* = 0.008) and leg press (*p* = 0.001) but was not significantly different between groups at six or 12 weeks. The bench press volume load for the 10-SET and 5-SET groups was 4879.40 ± 772.7 kg and 2407.0 ± 483.3 kg, respectively (*p* = 0.001). For the leg press, the volume load was 24,490.80 ± 4180.0 kg and 13,498.00 ± 2712.2 kg for the 10-SET and 5-SET groups, respectively (*p* = 0.001). 

### 3.1. Body Composition

For participants in the 5-SET group body mass and BMI increased at six weeks (*p* = 0.001 and *p* = 0.001 respectively) and 12 weeks (*p* = 0.001 and *p* = 0.001 respectively) when compared to their baseline values. There were no significant differences between groups for body mass and BMI at any time point (Table 2). There was a trend for an increase in body fat (%) for the 5-SET group at 12 weeks (*p* = 0.08), although no between-group differences were identified. No significant differences within or between groups were found for lean mass of total body, trunk, and arm at six or 12 weeks. Lean leg mass was found to significantly decrease from six to 12 weeks for the 10-SET group (*p* = 0.02), and no other differences were found at any other time point within or between groups.

Table 3 shows the ES with 95% CI within and between groups for the body composition outcomes. The ES for changes in body mass, body fat (%), total lean body mass, lean trunk mass, and lean arm mass within and between groups were trivial to small (ES = −0.02 to 0.31). A small ES was found for lean leg mass at six weeks for the 10-SET group (ES = 0.51), while a negative trivial ES was found for the 10-SET group at 12 weeks (ES = −0.19). Trivial to small ES were found for lean leg mass at six and 12 weeks for the 5-SET group (ES = 0.04 and 0.05) and between groups (ES = −0.30 and 0.18).

### 3.2. Muscle Strength

**Figure 1:**
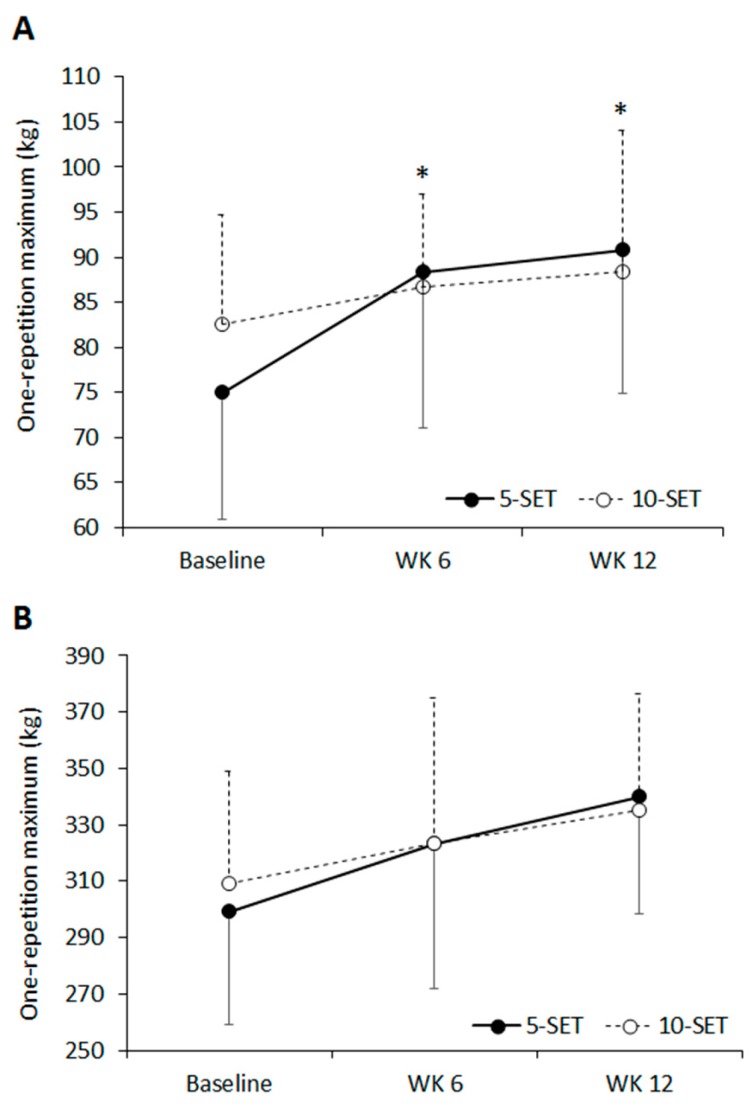
Upper and lower body muscle strength at baseline, six, and 12 weeks of training. One-repetition maximum at baseline, six weeks, and 12 weeks for the bench press (**A**) and leg press (**B**). Data are mean ± SD. * Significantly different to baseline for 5-SET group.

Figure 1 shows the absolute mean changes for the 1RM bench press and leg press. Bench press 1RM increased for only the 5-SET group at week 6 (*p* = 0.001) and 12 (*p* = 0.001) when compared to baseline values, while there were no significant increases in leg press 1RM at six and 12 weeks for any group. Also, there were no differences between the 5-SET and 10-SET groups in bench press and leg press at six and 12 weeks. 

As presented in Table 3, the 5-SET group showed moderate ESs for 1RM bench press at six and 12 weeks (ES = 0.84 and 1.05 respectively) when compared to baseline, whereas there were small ESs found at these time points for the 10-SET group (ES = 0.37 and 0.41 respectively) when compared to baseline. For the 1RM bench press, there were moderate ESs favoring 5-SET compared to 10-SET group at weeks 6 and 12 (ES = 0.65 and 0.63 respectively). Based on the ESs at six and 12 weeks, it was determined that a sample size of *n* = 18 would be required for the differences in 1RM bench press between groups to reach statistical significance. Small ESs for 1RM leg press were found for the 5-SET and 10-SET groups at six weeks (ES = 0.52 and 0.56 respectively) when compared to baseline, with a small ES found between groups favoring the 10-SET group (ES = −0.24). At 12 weeks, moderate ESs were found for the 5-SET and 10-SET groups (ES = 1.00 and 1.05, respectively), with a small ES found between groups favoring the 10-SET group (ES = −0.43). From this ES observed at 12 weeks, it was determined that a sample size of *n* = 36 would be required for the differences in 1RM leg press between groups to reach statistical significance.

## 4. Discussion

This study investigated the effect of a 12-week modified GVT program (10 sets method) compared to training performed with sets on the upper end of the range commonly used by resistance trainers (5 sets) on muscle strength and hypertrophy. Results showed there were no greater increases in muscular hypertrophy for the 10-SET compared to 5-SET group which does not support our original hypothesis. However, there was an unusual decrease in lean leg mass for the 10-SET group between weeks 6 and 12. In agreement with our other hypothesis, there was no difference in muscle strength between groups at the mid-point and following the intervention. Notably, for 1RM bench press moderate effect sizes in favor of 5-SET were found, whereas for 1RM leg press, there were small ESs in favor of 10-SET. Therefore, for muscle strength, the upper body may potentially respond more favorably to five sets compared to 10 sets whereas the opposite may occur for the lower body (10 sets being better than five sets). Future research is needed to elucidate the effect of resistance training volume (i.e., number of sets) on upper versus lower body muscle strength and hypertrophy. 

Previously it was thought that following a modified GVT program for six weeks duration might be too short a timeframe for significant muscle hypertrophy adaptations to be observed [19]. Therefore, extending the duration of the modified GVT program to at least 10–12 weeks might be required to allow for these muscle adaptations [21]. Contrary to our original hypothesis, the results of the present study suggest that 10 sets compared to five sets per exercise for 12 weeks does not lead to greater muscle hypertrophy. A significantly greater training volume was performed by the 10-SET compared to the 5-SET group, which was expected to accentuate factors implicated with muscle hypertrophy (e.g., metabolic stress, mechanical tension, muscle damage) [12]. The finding of 10 sets compared to five sets per exercise being ineffective for enhancing muscle mass may suggest a training volume upper threshold for muscle hypertrophy lies between 5–10 sets. This threshold may be closer to five sets, based on Krieger’s [9,10] observation of muscle hypertrophic adaptations being limited when training volume increases beyond 4–6 sets per exercise.

The finding of a significant decrease in leg lean mass for the 10-SET group from weeks 6–12 is difficult to explain. Other lean body mass measures remained either relatively stable (total body and arm lean mass) or increased (trunk lean mass) during this time period. However, there were slight reductions in body mass and body fat (%) from weeks 6–12 in the 10-SET group which may indicate that external factors such as physical activity levels or dietary practices may have changed for these participants during this period. Even though all participants were asked to refrain from any resistance training away from the supervised sessions, participants were not given instructions about other exercise. Additionally, participants were encouraged to increase their daily caloric intake through eating slightly bigger portions during meals throughout the study; however, dietary intake was not monitored throughout the intervention. Based on the 1RM leg press performances and relative training intensity (% 1RM) increasing for the 10-SET group during weeks 6–12, it appears that factors unrelated to the training intervention such as physical activity and diet may have contributed to the decrease in leg lean mass.

While there was a moderate effect size in favor of the 5-SET group for 1RM bench press, this same type of response was not found for 1RM leg press. The small effect size found favoring the 10-SET group for 1RM leg press may suggest that higher training volumes are more effective for increasing lower compared to upper body strength. Marshall et al. [6] found a greater increase in 1RM squat for eight sets compared to one set. However, Marshall et al. [6] found no differences in 1RM squat for one set versus four sets and for four sets versus eight sets, suggesting that strength gains from performing >4 sets are minimal. In the present study, the within-group effect sizes for 1RM leg press were ~0.5 and ~1.0 at six and 12 weeks, respectively, for both groups. Therefore, our findings suggest that the advantage of performing 10 sets compared to five sets for leg exercises would also be minimal.

The trivial effects for leg lean mass in the 5-SET group compared to the small effect found for the 10-SET group at six weeks suggests that a greater volume over a relatively short duration is effective for leg hypertrophy gains. These trivial effects on leg lean mass were maintained over the whole 12 weeks for the 5-SET group, whereas, as discussed previously, there was an unusual decrease for this measure in the 10-SET group. In contrast, for the upper body hypertrophy measure at each time point, there was a tendency toward slightly greater effects for the 5-SET compared to 10-SET group. This may also point toward a different response to resistance training volumes for muscles of the upper and lower body. A review by La Scala Teixeira et al. [25] provided evidence that high set volumes (≥3) are not significantly better than low set volumes (<3) in regard to upper body muscle hypertrophy. Also, there is some evidence that the upper body compared to lower body muscles have an increased hypertrophic capacity [26,27]. Potentially, the optimal number of sets for an exercise to maximize muscle hypertrophy may be greater for the lower body compared to upper body. Reasons suggested for the differences in muscle hypertrophy gains for these body regions include the upper body having a greater androgen receptor content [28] and the legs having a lower training response due to their greater everyday use [29]. Future research is needed to confirm whether the upper and lower body musculature responds differently to resistance training volumes.

Several limitations should be taken into account when interpreting the results of this study. As mentioned above, the diet of the participants was not monitored, which may have confounded the results. It does appear that differences in caloric intake between groups may have occurred based on increases in body mass and body fat (%) for the 5-SET group and no change for the 10-SET group. If the 5-SET group did have an increased caloric intake throughout the study, this may have influenced their muscle hypertrophy and strength gains. Diets that create a caloric surplus are more likely to optimize muscle hypertrophy through facilitating anabolic processes as well as supporting of training demands [30]. It should also be noted that increased muscle hypertrophy can be achieved in hypocaloric conditions, provided there is sufficient dietary protein [31]. However, if the 5-SET group did consume a more favorable diet to optimize muscle strength and hypertrophy, it seems unlikely that this would have changed the overall findings from the study due to the minimal gains observed in the 10-SET group. Also, unfortunately, physical activity outside the resistance training sessions needed to be controlled and monitored to reduce the risk of confounding the results.

Even though the participation rate was high (93%) and there were no adverse events reported, further measures to monitor fatigue and soreness could have been collected to enable more definitive conclusions to be made. Finally, due to the low number of participants (*n* = 12), results may have been different with a larger sample size. Also, it is possible that participants in either the higher or lower volume groups may have been more suited to a particular volume of training (i.e., responder versus non-responder) [6].

## 5. Conclusions

This study expands upon the findings of our six-week resistance training study that investigated the effect of a modified GVT intervention, or 10 sets method, versus five sets on muscle strength and hypertrophy. Results of our study suggest 10 sets compared to five sets per resistance exercise over 12 weeks is no more effective for increasing muscle strength and hypertrophy. However, the lack of statistical significance for measures in the present study is likely due to the small sample size. Based on the calculated effect sizes, there appears to be a possibility that strength and hypertrophy responses following higher training volumes may differ for upper and lower body muscles. Until further research is conducted to confirm this hypothesis, 4–6 sets per resistance exercise is advised to maximize muscle strength and hypertrophy for trained individuals.

## Figures and Tables

**Table 1:** Details of the resistance training intervention for the 5-SET and 10-SET groups.

Session 1			Session 2			Session 3		
Exercise	Load (1RM)	Sets x Repetitions	Exercise	Load (1RM)	Sets x Repetitions	Exercise	Load (1RM)	Sets x Repetitions
Flat Bench Press ^a^	60%	10 or 5 × 10	Leg Press ^a^	80%	10 or 5 × 10	Shoulder Press ^a^	60%	10 or 5 × 10
Lat-Pulldown ^a^	60%	10 or 5 × 10	Dumbbell Lunges ^a^	70%	10 or 5 × 10	Upright Row ^a^	60%	10 or 5 × 10
Incline Bench Press	70%	4 × 10	Leg Extensions	70%	4 × 10	Tricep Pushdowns	70%	4 × 10
Seated Row	70%	4 × 10	Leg Curls	70%	4 × 10	Bicep Curls	70%	4 × 10
Crunches	~RM ^b^	3 × 20	Calf Raisers	~RM ^b^	3 × 20	Sit-ups with twist	~RM ^b^	3 × 20

**^a^** 10 and 5 sets respectively for these exercises (10-SET, 5-SET). 1RM = one-repetition maximum; ~RM ^b^ = close to repetition maximum.

**Table 2:** Body composition at baseline, 6 and 12 weeks of training.

	5-SET	10-SET
**Body mass (kg)**		
Baseline	76.0 ± 16.4	83.3 ± 7.1
6 wks	79.2 ± 17.8 ^†^	84.8 ± 8.4
12 wks	80.0 ± 18.4 ^†^	83.9 ± 8.4
**Body mass index (kg/m^2^)**		
Baseline	24.2 ± 6.5	25.6 ± 2.9
6 wks	25.2 ± 6.7 ^†^	26.1 ± 3.2
12 wks	25.4 ± 6.7 ^†^	25.8 ± 3.4
**Body fat (%)**		
Baseline	18.2 ± 5.8	21.3 ± 9.5
6 wks	19.2 ± 5.9	21.1 ± 8.6
12 wks	19.5 ± 6.2 ^¥^	20.5 ± 9.0
**Lean body mass (kg)**		
Baseline	58.9 ± 9.6	62.5 ± 5.7
6 wks	60.6 ± 10.1	63.8 ± 5.9
12 wks	60.9 ± 10.0	63.5 ± 5.4
**Lean trunk mass (kg)**		
Baseline	26.7 ± 3.9	29.1 ± 3.0
6 wks	27.7 ± 4.4	28.8 ± 3.5
12 wks	28.0 ± 4.5	29.6 ± 3.2
**Lean arm mass (kg)**		
Baseline	7.9 ± 1.8	9.1 ± 0.4
6 wks	8.5 ± 2.1	9.2 ± 0.6
12 wks	8.5 ± 1.9	9.2 ± 0.8
**Lean leg mass (kg)**		
Baseline	20.3 ± 3.8	20.7 ± 1.9
6 wks	20.4 ± 3.5	21.7 ± 2.0
12 wks	20.5 ± 3.6	20.4 ± 1.6 ^‡^

Data are mean ± SD. ^†^ Significantly different from baseline (*p* = 0.001). ^‡^ Significantly different from week 6 (*p* = 0.02). ^¥^ Trend for significant difference compared to baseline (*p* = 0.08).

**Table 3:** Effect sizes for body composition and muscle strength outcomes.

	5-SET		10-SET		Between Groups		
	Effect Size	95% CI	Effect Size	95% CI	Effect Size	95% CI	Sample Size Required (*p* < 0.05)
**Body mass (kg)**							
6 wks	0.19	−0.62 to 1.00	0.19	−0.62 to 1.00	0.13	−1.00 to 1.26	-
12 wks	0.23	−0.58 to 1.04	0.08	−0.73 to 0.88	0.24	−0.90 to 1.38	104
**Body mass index (kg/m^2^)**							
6 wks	0.16	−0.65 to 0.97	0.15	−0.66 to 0.95	0.11	−1.02 to 1.25	-
12 wks	0.19	−0.62 to 1.00	0.06	−0.74 to 0.86	0.20	−0.94 to 1.33	-
**Body fat (%)**							
6 wks	0.19	−0.62 to 1.00	−0.02	−0.82 to 0.78	0.17	−0.96 to 1.31	-
12 wks	0.23	−0.58 to 1.04	−0.08	−0.88 to 0.72	0.28	−0.86 to 1.41	78
**Lean body mass (kg)**							
6 wks	0.17	−0.63 to 0.98	0.23	−0.58 to 1.04	−0.05	−1.08 to 1.18	-
12 wks	0.21	−0.60 to 1.01	0.19	−0.62 to 1.00	0.12	−1.01 to 1.25	-
**Lean trunk mass (kg)**							
6 wks	0.25	−0.57 to 1.06	−0.08	−0.88 to 0.73	0.32	−0.82 to 1.46	60
12 wks	0.31	−0.51 to 1.12	0.18	−0.63 to 0.99	0.19	−0.95 to 1.32	-
**Lean arm mass (kg)**							
6 wks	0.30	−0.51 to 1.12	0.27	−0.55 to 1.08	0.29	−0.85 to 1.43	72
12 wks	0.31	−0.51 to 1.12	0.22	−0.59 to 1.03	0.29	−0.84 to 1.43	72
**Lean leg mass (kg)**							
6 wks	0.04	−0.77 to 0.84	0.51	−0.34 to 1.36	−0.30	−0.84 to 1.43	68
12 wks	0.05	−0.76 to 0.85	−0.19	−1.00 to 0.62	0.18	−0.96 to 1.31	-
**1RM bench press (kg)**							
6 wks	0.84	−0.09 to 1.77	0.37	−0.46 to 0.87	0.65	−0.52 to 1.81	18
12 wks	1.05	0.05 to 2.04	0.41	−0.42 to 1.24	0.63	−0.53 to 1.79	18
**1RM leg press (kg)**							
6 wks	0.52	−0.34 to 1.37	0.56	−0.30 to 1.42	−0.24	−0.84 to 1.43	104
12 wks	1.00	0.02 to 1.98	1.05	0.05 to 2.05	−0.43	−0.71 to 1.58	36
